# Upper and Lower Urinary Tract Outcomes in Adult Myelomeningocele Patients: A Systematic Review

**DOI:** 10.1371/journal.pone.0048399

**Published:** 2012-10-31

**Authors:** Paul W. Veenboer, J. L. H. Ruud Bosch, Floris W. A. van Asbeck, Laetitia M. O. de Kort

**Affiliations:** 1 Department of Urology, University Medical Centre Utrecht, Utrecht, The Netherlands; 2 Department of Rehabilitation, University Medical Centre Utrecht, Utrecht, The Netherlands; S. G. Battista Hospital, Italy

## Abstract

**Background:**

The introduction of sophisticated treatment of bladder dysfunction and hydrocephalus allows the majority of SB patients to survive into adulthood. However, no systematic review on urological outcome in adult SB patients is available and no follow-up schemes exist.

**Objectives:**

To systematically summarize the evidence on outcome of urinary tract functioning in adult SB patients.

**Methods:**

A literature search in PubMed and Embase databases was done. Only papers published in the last 25 years describing patients with open SB with a mean age >18 years were included. We focused on finding differences in the treatment strategies, e.g., clean intermittent catheterization and antimuscarinic drugs versus early urinary diversion, with regard to long-term renal and bladder outcomes.

**Results:**

A total of 13 articles and 5 meeting abstracts on urinary tract status of adult SB patients were found describing a total of 1564 patients with a mean age of 26.1 years (range 3–74 years, with a few patients <18 years). All were retrospective cohort studies with relatively small and heterogeneous samples with inconsistent reporting of outcome; this precluded the pooling of data and meta-analysis. Total continence was achieved in 449/1192 (37.7%; range 8–85%) patients. Neurological level of the lesion and hydrocephalus were associated with incontinence. Renal function was studied in 1128 adult patients. In 290/1128 (25.7%; range 3–81.8%) patients some degree of renal damage was found and end-stage renal disease was seen in 12/958 (1.3%) patients. Detrusor-sphincter dyssynergy and detrusor-overactivity acted as adverse prognostic factors for the development of renal damage.

**Conclusions:**

These findings should outline follow-up schedules for SB patients, which do not yet exist. Since renal and bladder deterioration continues beyond adolescence, follow-up of these individuals is needed. We recommend standardization in reporting the outcome of urinary tract function in adult SB patients.

## Introduction

### Rationale

Historically, spina bifida (SB) has been a lethal condition. However, the introduction of adequate neurosurgical treatment (of both the spinal lesion and the hydrocephalus) and low-pressure management for the urinary tract now allow the majority of SB patients to survive into adulthood. Nevertheless, renal failure and lower urinary tract dysfunction continue to be a problem in SB patients during their lifetime. No systematic review on the long-term outcome in SB patients is available. Therefore, this literature study aims to systematically collect and analyse data on outcome of upper and lower urinary tract function in adult SB patients. Also, due to the increasing number of these patients, we also aimed to identify factors that might predict adverse urological outcome on the longer term so that recommendations can be made for follow-up.

### Research Questions

What is the outcome for bladder and kidney function in patients aged ≥18 years with open SB?

Which treatment regimens give the best outcomes with regard to renal and bladder functioning?

What are the risk factors for (ongoing) renal and bladder function deterioration in these patients?

## Methods

### Eligibility Criteria

Studies were included when the mean/median age at follow-up of patients was at least 18 years and presented details on urological outcomes in a standardized way. With regard to reporting characteristics, studies were found eligible when published in the last 25 years (February 17 1987–February 17 2012). Meeting abstracts and full-text articles were taken into consideration because of the known paucity of full-text papers on this subject.

### Data Sources

The search was performed on February 17 2012 using PubMed®/Medline and Embase™.

### Search

Synonyms for open SB (e.g. myelomeningocele, bifid spine) were combined with synonyms for bladder function and kidney function, and also with terms for diagnostic methods to assess these, e.g. urodynamics and renal scintigraphy. Only papers published in the last 25 years were included. No other limits were used; in this way the search was kept as sensitive as possible. The exact search (including search terms) is shown in Appendix 1.

### Study Selection

Results were exported to RefWorks® Version 2.0. Duplicates were deleted. Title screening was done by author PV. Abstract screening took place independently by PV and LdK. Screening results were compared and discrepancies were solved by discussion. Full-text screening (critical appraisal on both validity and relevance) was subsequently performed by PV with the assistance of LdK.

Only studies describing patients with a mean/medianage of at least 18 years, with open SB were included. Exclusion criteria were spinal cord lesions other than SB (e.g. traumatic spinal cord lesions), mean/median age <18 years, all forms of occult dysraphism, including tethered cord without SB. Studies had to describe either upper or lower urinary tract functioning; published articles and meeting abstracts were included. In studies describing a mixed neurological population, SB patients had to be analyzed separately from the other patients. If multiple subcohorts with different mean ages were described, only the subcohorts with a mean/median age >18 years were analysed. In studies describing renal function, only those papers were included in which renal functioning was defined by clear, standardized values, i.e. creatinine, glomerular filtration rate (GFR), renography or ultrasonography. In papers describing lower urinary tract functioning, standardized values were obligatory for inclusion, e.g., a clear definition of incontinence or a clear definition of urodynamic outcomes.

### Data Collection Process and Data Items

From all studies, the following data were extracted: number of patients, mean age (range), the treatment protocol followed, type and level of the lesion, presence of hydrocephalus, method of bladder emptying, previous surgery of the kidneys and lower urinary tract, renal functioning, urodynamic parameters (if provided), vesico-ureteral reflux, and use of antimuscarinics. Data were entered in a database using IBM Statistical Package for the Social Sciences (SPSS®) version 20.0.

Kidney function was classified according to the 2002 chronic kidney disease (CKD) classification of the The National Kidney Foundation Kidney Disease Outcomes Quality Initiative [1]. If possible, CKD stage was calculated. Patients who underwent renal transplantation in the past, but who had good renal function at the time of the study, were classified as CKD 5. CKD 5 is also referred to as ‘end-stage renal disease’ or ESRD. If mg/dL was used, this was re-calculated into µmol/L by dividing it by 0.018. Dryness or ‘complete continence’ was defined as completely dry both day and night with no need to wear pads. For lower urinary tract outcomes, the 2002 International Continence Society ‘Good Urodynamic Practice’ guidelines were used to standardize outcomes [2]. An attempt was made to categorize urinary incontinence (UI) into grades according to the number of UI episodes per time unit (day, week or month) if these time units were reported. Parameters of filling cystometry analysed were bladder capacity (in mL), bladder compliance, end-filling pressure, detrusor overactivity (DOA) and incontinence. End-filling pressure was defined as the pressure in cm H_2_O at the end of the filling phase, and bladder compliance was defined as Δ volume (mL)/Δ pressure (cm H_2_O) at the end of the filling phase. We correlated urodynamic parameters and outcomes of both upper and lower tract function. We analysed which treatment regimen preserved the urinary tract the best on the longer term.

### Summary Measures and Synthesis of Results

Means were primarily used to report outcomes in the various groups (e.g. different neurological level, treatments, urodynamics); differences in means (for treatment outcomes) and if possible odds ratios with *p*-values and 95% confidence intervals (for risk factors) were used. Harmonization of results according to the above-mentioned standards (CKD, good urodynamic practice) was done as much as possible to enable pooling of results.

### Bias in Individual and in All Studies

Bias in individual studies was noted. However, because no randomized controlled trials were available on this subject (see Results), certain biases could not be avoided. Survival bias was present in all studies, and this was taken into account.

## Results

### Search Results

**Figure 1:**
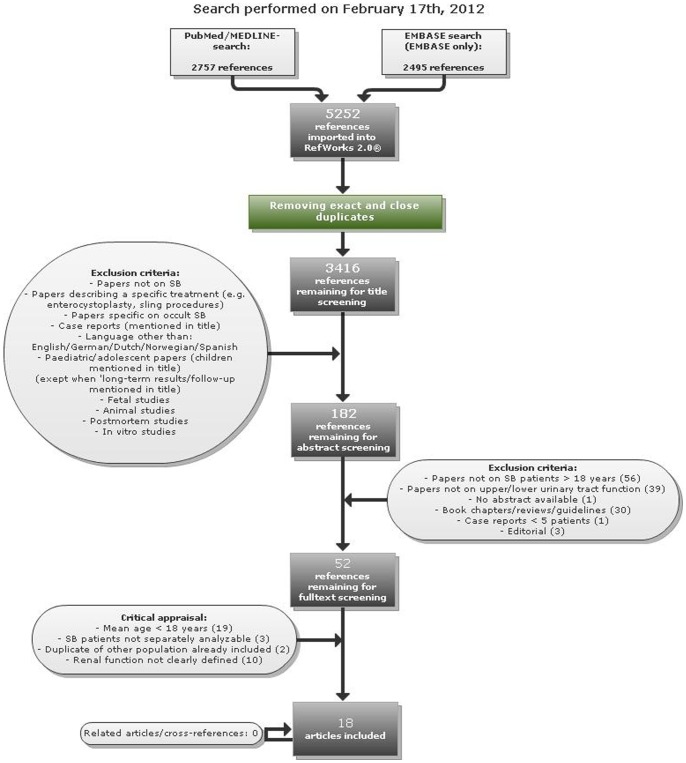
Search strategy and selection. The database search was performed on February 17 2012. For more details on the search strategy see the Materials and Methods section.

Search results are shown in Figure 1. A total of 18 studies (published between 1994 and 2011) were included describing 1564 patients [3]–[20]. Mean age of the included patients was 26.1 (standard deviation (SD) 6.0; range 2–74) years. Inclusion of patients <18 years could not be avoided if they were not separately analyzable, which was the case in 6 studies; 3 of these were meeting abstracts in which patients’ characteristics were only briefly described.

All included studies are retrospective cohort studies/case series with all inherent limitations (i.e. selection bias, information bias and no correction for confounders) except for one prospective study where patients were followed prospectively from the 1960 s, the so-called ‘Cambridge cohort’ [14]. Five of 18 included papers were published as meeting abstract only; the remaining 13 were published as full-text article.

### Baseline Characteristics: Type of Lesion, Level of Lesion, Hydrocephalus

In 13 studies (1234 patients), the type of SB was specified [3], [5]–[7], [10], [11], [13]–[17], [19], [20]. In 1132/1234 (93%) of the cases there was a form of open SB (predominantly myelomeningocele). Hydrocephalus was described in 6 studies (856 patients); 578/856 (67.5%) of these patients were described as having either hydrocephalus or a shunt for hydrocephalus [3], [5], [6], [11], [20]. Level of the lesion was described in 7 studies (430 patients): 1 cervical, 25 thoracic, 6 thoracolumbar, 40 lumbar, 44 lumbosacral, 38 sacral, and 5 had lesions that were described as ‘asymmetrical’. A total of 181 patients had a lesion ‘above L5’. In most cases data on lesion level and hydrocephalus were given for the entire study population and not specified for any subgroups.

### Bladder Emptying

Method of bladder emptying was described in most studies (15/18, *n = *1370) [3]–[7], [9], [11]–[17], [19], [20]. Clean intermittent catheterization (CIC) was by far the most frequently described method performed by 800/1370 (58.4%) of patients. Another 189/1370 (13.8%) voided normally, whereas 144/1370 (10.5%) of patients were described as having incontinent diversions. In 5.9% (81/1370) of patients complete incontinence was described and 59/1370 (4.3%) had an indwelling catheter. Emptying by Crede’s manoeuvre was utilized by 40/1370 (2.9%). Another 25/1370 (1.8%) used a combination of CIC, Crede and spontaneous voiding. The bladder emptying method of the remaining 32 patients (2.3%) is unknown.

### Surgery

Surgery was described in 12 studies which included 1272 patients [5]–[7], [10]–[15], [17], [19], [20]. Enterocystoplasty was performed most frequently in 227/1272 patients (17.8%), and 146/1272 (11.5%) had an ileal conduit. A sling procedure was performed in 55/1272 (4.3%), whereas 53/1272 (4.2%) had an artificial sphincter implanted to achieve continence. In 53/1272 (2.2%) patients some kind of anti-reflux procedure was performed and 14/1272 (1.1%) had undergone nephrectomy. A sphincterotomy/bladder neck incision, pouch formation and detrusorectomy were described in 15, 9 and 4 patients, respectively. Not all studies specified the whole range of available surgeries, some only mentioned the number of diversions and nephrectomies.

### Urodynamics

Urodynamic outcomes were reported in 7 studies describing 296 patients: 18.9% of 1564 included patients; mean age 29.3 (range 16–69) years) [6], [7], [9], [10], [15]–[17], [19]. Complete urodynamic data including capacity, pressures and compliance were rarely available, and only in a subgroup of patients. Mean bladder capacity (reported in two studies [7], [15]) was 388 (range 293–485; SD 136) mL. Mean end-filling pressure, described in the same two studies, was 20.7 (range 18–28) cm H_2_O. Four studies reported the number of patients with end-filling pressures >40 cm H_2_O: 24/178 (13.5%) [6], [9], [10], [15]–[17], [19].

Bladder compliance was reported in only 3 studies [7], [15], [17]. One study [15] presented with numeric (continuous) values; in another study [7] compliance was reported categorically, but without cut-off values. In a third series [17] only the number of patients with poor compliance was mentioned (again, without a clear definition of ‘poor’). Altogether, good/normal compliance was reported in 22/32 (68.8%) patients, borderline compliance in 4/32 (12.5%) patients, and poor compliance in 44/130 (33.8%) patients. One study [16], [19] reported the number of patients with detrusor sphincter dyssynergy (DSD): 9/40 (22.5%) patients had DSD. DOA was mentioned in 3 studies [6], [15], [17], [19], with 40/162 (24.7%) patients having DOA. Thorup *et al.* mentioned DOA and DSD, but only at inclusion during childhood for the prediction of renal outcomes in adulthood and not at the time of study [19].

### Vesico-ureteral Reflux (VUR)

There is a paucity of reported data on VUR in adult SB patients. Three studies (*n* = 126 patients) described the prevalence of VUR [6], [9], [19]. Only one study [6] specified the degree of reflux. Some degree of VUR (grade I-V) was seen in 26/126 (20.6%) patients.

### Incontinence

Twelve studies were selected on incontinence [3]–[7], [12]–[15], [19]–[21]. Results are shown in Table 1. Definitions of incontinence were different in each study. Most authors are consistent regarding the definition of ‘complete dryness’: no involuntary urinary loss. Surprisingly, urodynamic parameters were not correlated with continence status.

**Table 1:** Urinary incontinence (UI); studies in chronological order.

First author (year)	*n*	Type SB	Age in years, mean (±SD) (range)	Complete dryness (%)	Complete dryness pertype of bladderemptying method (%)			Complete dryness relatedto neurological status (%)			Type/degree of UI	Occurrence (%)^a^	
**Herschorn (1994)** [7]	20^b^	Mixed	33 (25–45)	**17 (85%)**	NS			NS			Night-time UI Stress UI 1x/day	1	(5%)
												2	(10%)
**Malone (1994)** [12]	109	Mixed	23.5 (9–47.8)	**51 (47%)**	UD−	20/28	(71%)	NS			NS	NS	
					UD+	31/89	(38%)						
					CIC+	7/23	(30%)						
					Void	15/21	(71%)						
					IC	5/16	(31%)						
					Crede	4/10	(40%)						
**Peeker (1997)** [15]	12	Open	23.3 (20–31)	**1 (8%)**	CIC	1/12	(8%)	NS			Night-time UI	2	(17%)
											UI both day and night	9	(75%)
**McDonnell (2000)** [13]	193	Mixed	27.6 (range/SD N/A)	**16 (8.3%)**	NS			NS			Urgency/frequency	31	(16.1%)
											Completely dependent on pads	8	(4.1%)
**Glott (2001)** [6]	33^c^	Open	28 (16–46)	**3 (9%)**	NS			NS			UI ≥1x/day	20	(60%)
											UI <1x/day	10	(30%)
											Stress UI	27	(82%)
											Non-stress UI	24	(73%)
**Bowman (2001)** [3]	71	Open	21.7 (19.4–24.8)	**57 (80.3%)** d	CIC+	50/60	(83%)*	NS			UI 50% of time^e^	6	(10%)
					CIC−	7/11	(63%)*				UI 50–100% of time^e^	4	(7%)
											UI 100% of time^e^	9	(15%)
**Verhoef (2005)** [20]	179	Mixed	20.4 (16–25; ±3.0)	**70 (39.9%)**	NS			HC+	35/119	(29.4%)	UI > once a month	HC+	(70.6%)
								HC−	35/60	(58.3%)		HC−	(41.7%)
								L5↑	43/141	(31.2%)		L5↑	(68.8%)
								S1↓	26/38	(68.4%)		S1↓	(31.6%)
								IQ ≤70	8/23	(34.8%)		IQ ≤70	(65.2%)
								IQ >70	58/146	(40%)		IQ >70	(60%)
								ambu−	19/70	(27.1%)		Ambu−	(72.9%)
								ambu+	51/109	(46.8%)		Ambu+	(53.2%)
**Lemelle (2006)** [11]	398^e^	Open	22.1 (±7.9)	**136 (34%)**	NS			NS			UI rarely	33	(8%)
											UI occasionally	69	(17%)
											UI frequently	59	(15%)
											UI continuously	101	(25%)
**Oakeshott (2007)** [14]	50	Open	38 (34-31)	**11 (22%)**	NS			PS +	10/23	(43%)	NS	NS	
								PS −	1/27	(4%)			
**Chan (2010)** [4]	27	Mixed	23 (18–28)	**6 (22%)**	NS			NS			No bladder control	3	(11%)
													
**Thorup (2011)** [19]	52	Open	29^g^ (19–41)	**25 (48%)**	NS			NS			NS	NS	
													
**Cox (2011)** [5]	24	Mixed	26.8 (19–66)	**13 (44.2%)**	NS			NS			NS	NS	
													
**TOTAL**	**1192**		**23.8 (9–66)**	**449 (37.7%)**	**Cannot be pooled**			**Cannot be pooled**			**Cannot be pooled**		

Ambu = ambulant; CIC = clean-intermittent catheterisation; Crede = manual expression to empty the bladder; IC = indwelling catheter; HC = hydrocephalus; L = lumbar; MMC = myelomeningocele; NS = not specified; PS = perineal sensation; S = sacral; UD = urinary diversion; UI = urinary incontinence; void = normal voiding.

a If no subgroup is mentioned, this figure applies to the entire cohort.

b 20 patients with incontinent diversion after undergoing undiversion;

c all patients with intact bladder (i.e., no UD);

d dry was defined as: ‘continent most of the time’;

e these figures are only given for patients performing CIC;

f patients without UD;

g exemption; only median age is stated, whereas in all other studies, mean age is given.

A total of 1192 patients were included in these 12 studies. Total continence was achieved in 449/1192 patients (37.7%; range 8–85%). Treatment modalities used in these patients were very heterogeneous and therefore not easy to pool. The success of treatment modalities was discussed in studies by Lemelle *et al.* [11]; they showed that continence outcome (from the patient’s perspective) was better after surgical intervention. From other studies, no clear conclusions could be drawn, given the wide range of success rates in the different therapeutic subgroups. Whereas in one series the CIC cohort had mainly favourable results (Bowman *et al.* [3]), in other cohorts (Peeker *et al.* [15], Malone *et al*. [12]), those on CIC were not dry. Two studies found a relation between level of the lesion and (in)continence [14], [20], whereas in another study this could not be confirmed [15]. Patients with hydrocephalus were found to have more incontinence, although in a multivariate model only level of the lesion remained a significant risk factor for incontinence (*p* = 0.016; OR 2.749) [20].

### Upper Tract Functioning

Details on the results of the 13 studies describing renal functioning are presented in Table 2 [5]–[11], [13], [15]–[19]. A total of 1128 patients were included in these studies. Creatinine levels (and calculation of clearance) were most often used to determine renal impairment. When taking all degrees of renal failure together measured by serum creatinine/(e)GFR or imaging studies, a total of 290/1128 patients (25.7%; range 3–81.8%) had some degree of renal failure. A majority of 838/1128 (74.3%, range 8.2–97%) had well preserved kidneys.

**Table 2:** Renal function adult studies (MMC patients) in chronological order.

First author(year)	*n*	Type SB	Age in years, mean (±SD) (range)	Impaired kidney function (*n,* %)	Defined by	Treatment*	Factors associated with renal deterioration	
**Herschorn (1994)** [7]	20	Mixed	27 (19–39)	**9/20 (45%)**	IVP changes; serum creatinine	Ileal conduit	• Not described.	
				CKD N/A				
**Peeker (1997)** [15]	12	Open	23.3 (20–31)	**5/12 (42%)**	Serum creatinine	CIC and AMD since age12 years	• Low compliance (2/5; 40%) (<10 ml/cm H_2_O)	
				CKD 1∶3 (25%)			• DOA (1/5; 20%)	
				CKD 2∶2 (17%)				
**Persun (1999)** [16]	40	Open	Mean age not given; range18–37	**20/40 (50%)**	Serum creatinine(≥83 µmol/L)	N/A	• DSD (6/20; 30%)	
				CKD N/A	or hydronephrosis on US		• Elevated EFP (6/20; 30%) (>40 cm H_2_O)	
**McDonnell** **(2000)** [13]	181^a^	Mixed	28.1 (14–59)	**89/181 (49.2%)** b	Serum creatinine, DMSA-scanning	N/A	• Not described.	
				CKD 1–4∶84 (46.4%)	or ultrasound(hydronephrosis)			
				CKD 5∶5 (2.8%)				
**Glott (2001)** [6]	51	Open	30 (16–46)	**19/51 (37%)**	^99m^Tc-DTPA	Mixed	• Pouch/diversion (8/18; 44% GFR below expected;	
				CKD N/A	(ref.: expected GFR for age)		vs. 11/33 (34%) without diversion/pouch).	
**Kessler (2006)** [10]	22	Open	20 (12–30)	**3/22 (13.6%)**	DMSA uptake	CIC and AMD	• Late start of CIC/AMD	
				CKD N/A				
**Lemelle (2006)** [11]	421	Open	21 (±7.9) (Medically treated )	**13/421 (3%)**	Serum creatinine	Mixed	• Urinary diversion (13/13; 100%)^c^	
			23 (±7.6) (Surgically treated)	CKD 1–4∶9 (2%)				
				CKD 5∶4 (1%)				
**Reyblat (2010)** [17]	98	Open	38 (20–69)	**7/98 (7.1%)**	Serum creatinine(>61.1 µmol/L)	Mixed	• Worse renal function in group who underwent bladder	
				CKD N/A			• surgery (N.S.)	
**Swallow (2010)** [18]	92	N/A	19.6 (2–38)	**41/92 (45%)**	Serum creatinine & GFR	CIC and AMD since birth	• Age (patients with CKD 2 older than CKD 3)	
				CKD 1,2,4∶11 (12%)				
				CKD 3∶29 (32%)				
				CKD 5∶1 (1%)				
**Hsu (2010)** [8]	80	N/A	21.8 (3–74)	**48/80 (60%)**	Creatinine-EDTA (clearance)	Mixed	• Not described.	
				CKD 1∶7 (9%)				
				CKD 2∶31 (39%)				
				CKD 3∶8 (10%)				
				CKD 4∶1 (1%)				
				CKD 5∶1 (1%)				
**Thorup (2011)** [19]	52	Open	29 (19–41)^e^	**15/52 (29%)**	Creatinine-EDTA (clearance)	Mixed	• DSD (5/8; 63%) (childhood urodynamics)	
				CKD 1∶7 (14%)			• DOA (5/8; 63%); *p = *0.04 (childhood)	
				CKD 2–4∶7 (14%)			• RUTIs (8/8; 100%) (childhood)	
				CKD 5∶1 (2%)			• Elevated LPP (6/8; 75%) (childhood)	
							• Reflux (5/8; 63%) (childhood)	
**Inoue (2011)** [9]	22	N/A	22 (18–36)	**18/22 (81.8%)**	Unilateral DMSA uptake <15%	CIC before age 6 years (45.6%)	• LPP (*p*<0.05)	
				11 (bilateral nephropathy)	Bilateral DMSA uptake <30%	CIC after age 7 years	• Age	
				7 (unilateral nephropathy)		(55.4%)	• Febrile UTIs	
				CKD N/A			• No correlation with early start of CIC	
**Cox (2011)** [5]	24	Mixed	26.8 (19–66)	**3 (12.5%)**	Serum creatinine	N/A	• Not described.	
				CKD 1–4∶3 (12.5%)				
				CKD 5∶0 (0%)				
**TOTAL**	**1128**		**25.0 (3–74)**	**CKD 1–5∶290/1128 (25.7%)**	**Cannot be pooled**	**Cannot be pooled**	**Cannot be pooled**	
				**ESRD (CKD 5): 12/958** f **(1.3%)**				

AMD = antimuscarinic drugs; CKD = chronic kidney disease; N/A = not available; DOA = Detrusor overactivity; DSD = detrusor sphincter dyssynergia; ESRD = end-stage renal disease; GFR = glomerular filtration rate; LPP = leak point pressure; Mixed: occult and open SB; VUR = vesico-ureteral reflux; rUTIs = recurrent urinary tract infections; N.S. = not significant.

a in 12/193 patients renal function was unknown;

b no reference values of renal function;

c no urodynamic data available;

e **exemption; only median age is stated, whereas in all other studies, mean age is given.**

f other denominator; only studies with known CKD’s are taken into account for this figure (see Results-section for more details).

Renal function continued to deteriorate after childhood. In one study, 2/8 children with normal renal function during childhood developed renal damage during puberty [19].

CKD stages were directly available in two studies which included a total of 172 patients [8], [18]. In another 8 studies with 786 patients [5], [9]–[11], [13], [15], [16], [19] CKD stages could be calculated; in one article with 12 patients with 100% accuracy [15]. For the remaining 3 articles, CKD stages were unknown and could not be extracted from the data [6], [7], [17]. In 10 studies describing CKD stages, 958 patients were included. End-stage renal disease (ESRD, CKD 5) was described in 12/958 (1.3%) patients, and 694/958 patients (72.4%) were reported to have well preserved kidneys (CKD 0). The remaining 252/958 (26.3%) patients had renal dysfunction between CKD 1 and 4. Most patients had stage 1–3 CKD (Table 2).

#### Risk factors for renal deterioration

Various authors have identified risk factors for the development of CKD. DSD, DOA, high leak point pressure and high intravesical pressures are among the urodynamic risk factors (see Table 2) [9], [19]. Additionally, patients who underwent surgery of the lower urinary tract more often had upper urinary tract deterioration. Unfortunately, data could not be pooled, precluding the elimination of bias. In two studies, urinary tract infections during childhood were associated with renal deterioration later in life [9], [19].

Not all studies report on the different strategies and therapies used to preserve the urinary tract. Most authors present cohorts that were treated with a variety of regimens, ranging from early intervention with CIC and antimuscarinic agents, to early surgical intervention by urinary diversion, or no urological treatment at all. In most studies age of starting CIC was not mentioned, accept in two articles where patients started at a mean age of 11 and 12.8 years, respectively [10], [15]. Starting early with a pro-active regimen proved to be better in one study [10]: patients who started treatment after the age of 10 years had more upper tract deterioration than patients who started between 0 and 2 years of age (14% vs. 1%, *p* = 0.012). Also, the late treatment group underwent significantly more surgery of the urinary tract than the young starting group (59% vs. 15%, *p* = 0.0002). In another study the time of starting CIC was not predictive for renal function [9].

## Discussion

Nowadays, an increasing number of patients with SB survive into adulthood. This is the first systematic literature study to summarize all the available data on the outcome of upper and lower urinary tract in adult SB patients.

### Survival into Adulthood

In this review, survival into adulthood could be an important cause of bias. Patients with very poor renal function, hydrocephalus and shunt dysfunction and high level of the lesion will probably be underrepresented. This impedes adequate analysis of risk factors for kidney and bladder deterioration. Table 3 summarizes studies reporting on survival data into adulthood in relatively large SB cohorts from the 1970s onwards [14], [22]–[28]. The authors of the Cambridge cohort (a prospectively followed cohort without survival bias), calculated the annual risk of death to be about 1% per year between the 5^th^ and 30^th^ year of life [29]. In the same series, the number and cause of deceased patients were accurately reported, with 17/60 patients dying before the age of 35 years because of renal failure [24]. Assuming that nowadays two thirds to three quarters of myelomeningocele patients survive into adulthood, we can make a (rough) estimation of the survival bias playing a role in this review. Given the fact that ESRD is the cause of death in 8.9–28.6% of all cases and survival has been 54–88%, the number of patients that have probably died from renal failure in the total 958 patients (for whom CKD stages were available) would be somewhere between 11 and 233 patients. This is considerably more than the 12 patients with ESRD we found without taking this bias into account.

**Table 3:** Survival in spina bifida patients.

First author (year)		Survival rates	Main causes of death (frequency)
**Barden** [22] **(1975)**	*n = *63	68% beyond 6 years	Meningitis (8/34); 23.5%
		54% beyond 20 years	Unknown (13/34); 38.2%
			Hydrocephalus (2/34); 5.9%
			**Renal failure (7/34); 20.6%**
			Pneumonia (1/34); 2.9%
			Other (3/34); 8.8%
**Bowman** [3] **(2001)**	*n = *118	75% into adulthood	Hindbrain dysfunction (13/28):
			Shunt dysfunction (N/A)
			**Renal failure (0/28): 0%**
			
**Hunt** [24] **(2003) (‘Cambridge cohort’)**	*n = *117	44% until 35 years	Cardiorespiratory (19/63); 30.2%
		(range: 32–38 years)	**Renal failure (18/63); 28.6%**
			Hydrocephalus (10/63); 15.9%
			CNS infection (10/63); 15.9%
			Convulsions (2/63); 3.2%
			Inhaled vomit (2/63); 3.2%
			Sudden infant death (1/63); 1.6%
			Trombocytopenic purpura (2/63); 3.2%
**Davis** [23] **(2005)**	*n = *904	54% beyond 16 years*	N/A
		85% beyond 16 years†	
**Oakshott** [14] **(2007) (‘Cambridge cohort’)**	*n = *117	70% until 38 years(with perineal sensation)	0% renal deaths (with perinal sensation)
		32% until 38 years (without perineal sensation)	23% renal deaths (without perineal sensation)
**Oakshott** [25] **(2008) (‘Cambridge cohort’)**	*n = *1041	38% until 50 years	N/A
			
			
**Oakshott** [26], [47] **(2010, 2012)** **(‘Cambridge Cohort’)** ‡	*n = *117	65% beyond 5 years	(See Hunt *et al.* 2003)
		42% beyond 40 years	
			
			
**Tennant** [28] **(2010)**	*n = *541	71% into adulthood	N/A
		(56% with hydrocephalus; 88% without hydrocephalus)	
**Roach** [27] **(2011)**	*N = *149	82% beyond 10 years	General infection (15/45); 33.3%
		70% beyond 30 years	Hydrocephalus (7/45); 15.6%
			Heart failure (5/45); 11.1%
			**Renal failure (4/45); 8.9%**
			Unknown (14/45); 31%

If ‘survival into adulthood’ is given, the exact ages were not specified in the article.

* Patients treated before 1975.

† Patients treated after 1975.

‡ Two different papers, same study.

N/A = not available

### Lower Urinary Tract/Incontinence

We found that the presence of UI in adulthood seems to be dependent on the type of lower urinary tract management, the presence of hydrocephalus, the neurologic level of the lesion and the presence of perineal sensation. The heterogeneity of the SB population and lack of protocolized treatment hinders generalization of data and recommendations on treatment. Urodynamic results, which can give a good impression of actual bladder function, are reported very inconsistently and are not complete. Assuming that bladder function may change over time and that bladder function influences renal condition and continence state, it is striking that urodynamic data are so poorly reported. The outcomes of surgery are discussed in detail only by Lemelle *et al.* and only very briefly mentioned by other authors [11]. Regarding surgery for incontinent SB patients they conclude that each patient should be approached as an individual and that no standard solutions exist. Therefore, no generalized statements on this topic can be made.

Although high rates of incontinence were found in some studies, it remains unclear how relevant this is for the patient. The relation between incontinence and quality of life in this patient group is not well established, but has been studied by Verhoef *et al.* [20] and Lemelle *et al.* [21]. The latter showed no strong relationship between health-related quality of life and UI while Verhoef *et al.* reported that a majority of patients (76/109; 69.7%) regarded UI as a problem influencing quality of life.

There are indications that bladder function deteriorates during puberty [30], although the exact cause is unknown. Proposed reasons are 1) adolescent rebellion against the discipline of CIC or medication, and/or 2) increased bladder outlet resistance due to growth of the prostate and estrogenization of the female urethra, or 3) tethering of the spinal cord [30]. A study by Almodhen *et al.* in a group of 37 patients showed that total cystometric bladder capacity, maximum detrusor pressure and detrusor leak point pressure significantly increased after puberty [31]. Symptomatic tethered cord occurs in 2.8–32% in all patients who underwent surgical myelomeningocele repair in the neonatal period [32]. The phenomenon of re-tethering later in life has not been widely investigated in adults. Given the growing number of adult SB patients, more knowledge will become available on deterioration of bladder function during puberty. However, current evidence (although scarce) indicates that patients cannot be discharged from follow-up after they have reached adulthood.

### Renal Functioning

Of all SB patients, 90% are born with a normal upper urinary tract; without any treatment, 50% will deteriorate if not treated appropriately [33]. Renal failure is still the leading cause of death in older SB patients, with renal insufficiency responsible for 30% of all deaths [34]. McGuire et al. published an important paper in the early 1980s describing increasing risk for the upper tract with bladder pressures exceeding 40 cm/H_2_O [35]. Lawrenson *et al.* showed that SB patients have a nine-fold higher risk of developing renal failure compared to their healthy peers [36]. The causal factors of renal failure in adult SB patients are usually treatable: high bladder pressure with or without VUR, urolithiasis, and, less frequently, ureteric obstruction in urinary diversions. Bladder function as a predictor of renal damage has been formalized by Galloway *et al.* [37]. A ‘hostility score’ including high-leak point pressures, bladder contractility, VUR and DSD was used as a predictive tool for renal outcome. This score has not been validated in adult patients.

The way of assessing kidney function in SB patients is an ongoing topic of debate. Serum creatinine often gives an overestimation of GFR, because of relatively low lean body mass in SB patients. Among others, cystatin-c has been proposed as a more sensitive alternative to measure renal function, being independent from muscle mass. However, in the only study conducted in SB patients by Abrahamsson *et al.* [38], cystatin-c was not able to predict slightly to moderately reduced renal function when compared to the gold standard (according to these authors) chromium-51-EDTA. The latter has also been used in some of the included studies (Table 2) as have other nuclear isotopes. Ultrasonography is an excellent screening tool for renal outline: it is inexpensive, non-invasive and without radiation exposure, but may be challenging in patients with severe scoliosis. The sensitivity of renal ultrasound to detect small scars was poor in one large series of children comparing ultrasonography with dimercaptosuccinic acid renal scintigraphy: 5.9% for the detection of focal renal scarring and 47.2% for the detection of diffuse renal scarring [39]. Renal scintigraphy is a good measure for renal function and scarring, but has the drawback of (minor) radiation exposure [40]. For interpretation of the results, it is important to standardize the reporting of renal functioning. In addition, a flow chart for diagnostic steps in case of suspected renal impairment is recommended.

The McGuire principle (as discussed above) is the basis for the policy to start early treatment with antimuscarinics and CIC. There is evidence that with an early regimen renal function can be spared in childhood [41], [42]. However, results in adulthood are somewhat contradictory. Substantiated arguments for a lifelong regimen of CIC and antimuscarinics are indispensable.

Renal function may deteriorate during puberty and may continue to worsen during adulthood, as supported by several small studies [9], [16], [19]. This can also be linked to the progressively deteriorating bladder function. Decrease in renal function is probably related to changing bladder function, but other factors such as hormonal alterations, increase in body weight and blood pressure may play a role. Larger cohort studies with multivariate analysis are needed to elucidate this topic.

The influence of surgery is only very briefly described in most reviews, with a tendency for worse renal functioning in those patients that underwent bladder surgery [6], [17]. The question remains whether the indication for bladder surgery was deterioration of renal function, or whether renal impairment was the result of bladder surgery (so-called ‘confounding by indication’); this issue has not been addressed.

Another threat for renal function in adult SB patients are urinary tract infections (UTIs). In 2 studies UTIs were associated with renal deterioration. Although our search yielded articles on the management/prevention of UTIs in SB patients, none matched our inclusion criteria. However, it is useful to briefly discuss three of these papers. A survey was held among SB clinics in the USA comprising circa 8000 patients, with two-thirds being paediatric clinics; results suggested that the management of bacteriuria was not protocolized, with every clinic having a different approach [43]. A 2009 study pointed out the high costs of hospitalizations for UTIs among adult SB patients, and the amount of money that might be saved with better implementation of protocolized preventive care for UTIs and bacteriuria [44]. Simply describing prophylactic antibiotics might not be an appropriate form of prevention. Apart from the emergence of multi-resistant bacteria, a recent study (in children) showed that stopping prophylaxis does not significantly increase the number of UTIs and significant bacteriuria [45]. Much more research is needed on this subject to determine the best practice for dealing with UTIs.

### Strengths and Limitations of this Review

This is the first systematic review on adult SB patients and upper and lower tract functioning. Shortcomings of this review are that the quality of the evidence is weak. Methodology of the included studies was generally poor; not even two papers used the same standards. This was recognized earlier by Ruffion et al. in a small systematic review on neurogenic overactive bladder [46]. Baseline characteristics (e.g. level of the lesion and hydrocephalus) were poorly described, whilst there is evidence that these factors are relevant for the urological outcome of SB patients. It was inevitable that some younger patients would be included, but this was only done when the majority of patients were aged ≥18 years. However, most studies are relatively recent and, thanks to our broad search, all relevant recent papers on the subject were included. It is clear that more research on the subject of long-term outcomes is needed.

### Recommendations

Standardization of reporting both upper and lower urinary tract function in SB patients is urgently needed. We recommend that every study describes the type and level of the lesion, presence of hydrocephalus, and urological treatment regimen, including previous surgery. In addition we recommend use of standardized terminology of the International Continence Society for outcomes of the lower urinary tract and urodynamics, and to use the CKD classification when reporting renal outcome. It is difficult to make recommendations based on the quality of evidence in this review. However, it is advisable to perform urodynamics at a certain interval (every 2–3 years) to note subclinical changes in bladder behaviour. Also, it might be advisable to perform a routine ultrasonography every year at follow-up, since this is an inexpensive, non-invasive and reliable screening method for gross renal changes and is much more reliable than serum creatinine. If renal changes are suspected at (in-office) performed ultrasound, more costly nuclear tests might be performed. However, to support these recommendations, more studies are needed.

### Conclusion

The urinary tract in SB patients evolves continuously during lifetime, with a continuing threat of high bladder pressures and renal damage, both during and after puberty. Level of the lesion and intravesical pressures seem to correlate with renal impairment. The percentage of patients with ESRD is low (1.3%), but survival bias may play a role. Incontinence was found in more than half of the patients, with unclear significance for quality of life. In adults, the long-term benefit of pro-active treatment with clean intermittent catheterization and antimuscarinics has yet to be proven. This review is also a plea for increased standardization in the reporting of urological outcomes in adult SB patients, since meta-analysis is not feasible at present.

## Supporting Information

Appendix S1 Search strategy
(DOCX)
