# Supplementary material for: Upper and Lower Urinary Tract Outcomes in Adult Myelomeningocele Patients: A Systematic Review
Source: PLoS One. 2012 Oct 31;7(10):e48399. doi: 10.1371/journal.pone.0048399 (PMC3485227; doi:10.1371/journal.pone.0048399)
Supplement: Appendix S1 — Search strategy (DOCX) [file pone.0048399.s001.docx]

# Appendix 1

**Search done on 17 February 2012**

PUBMED:

#1: **("spina bifida"[Title/Abstract] OR “spinal dysraphism”[Title/Abstract] OR myelomeningocele[Title/Abstract] OR SB[Title/Abstract] OR meningomyelocele[Title/Abstract] OR MMC[Title/Abstract] OR meningocele[Title/Abstract] OR “meningocystocele”[Title/Abstract] OR "bifid spine"[Title/Abstract] OR "spinal bifida"[Title/Abstract] OR "neural tube defects"[Title/Abstract] OR (Spinal dysraphism[MeSH]) OR myelodysplasia[Title/Abstract] NOT ("migrating motor complex"[Title/Abstract] OR mitomycine[Title/Abstract] OR mitomycin[Title/Abstract] OR "bone marrow"[Title/Abstract] OR haematology[Title/Abstract] OR leukaemia[Title/Abstract] OR "folic acid"[Title/Abstract] OR folate[Title/Abstract]))** **AND (** #2 **(“urinary incontinence”[Title/Abstract] OR “incontinence”[Title/Abstract] OR “enuresis”[Title/Abstract] OR “urinary loss”[Title/Abstract] OR “urine loss”[Title/Abstract] continence[Title/Abstract] OR “urinary continence”[Title/Abstract] OR "Urinary Incontinence, Urge"[Mesh] OR "Urinary Incontinence"[Mesh] OR "Urinary Incontinence, Stress"[Mesh] OR "Diurnal Enuresis"[Mesh] OR "Nocturnal Enuresis"[Mesh] OR dry[Title/Abstract] OR continent[Title/Abstract] OR incontinent[Title/Abstract] OR dribbling[Title/Abstract] OR dribble[Title/Abstract] OR dryness[Title/Abstract] OR wet[Title/Abstract] OR wetting[Title/Abstract] OR bed-wetting[Title/Abstract]) OR** #3 **(“lower urinary tract”[Title/Abstract] OR “bladder function”[Title/Abstract] OR “bladder capacity”[Title/Abstract] OR urodynamics[Title/Abstract] OR “urodynamic”[Title/Abstract] OR “bladder pressure”[Title/Abstract] OR “leak point pressure”[Title/Abstract] OR “bladder pressure”[Title/Abstract] OR “intravesical pressure”[Title/Abstract] OR “intravesical”[Title/Abstract] OR vesical[Title/Abstract] OR “detrusor sphincter dyssynergia”[Title/Abstract] OR “dyssynergia”[Title/Abstract] OR “dyssynergic”[Title/Abstract] OR bladder[Title/Abstract] OR “urinary bladder”[Title/Abstract] OR “pressure flow studies”[Title/Abstract] OR “pressure flow study”[Title/Abstract] OR PVR[Title/Abstract] OR “post void residual”[Title/Abstract] OR “post void residue”[Title/Abstract] OR “post-voiding residue”[Title/Abstract] OR urodynamic[MeSH]) OR** #4 **("Renal Insufficiency”[MeSH] OR "Renal Insufficiency, Chronic"[MeSH] OR "Kidney Failure, Chronic"[MeSH] OR kidney[Title/Abstract] OR CKD[Title/Abstract] OR ESRD[Title/Abstract] OR dialysis[Title/Abstract] OR “renal replacement”[Title/Abstract] OR “renal substitution”[Title/Abstract] OR “chronic kidney disease”[Title/Abstract] OR renal[Title/Abstract] OR kidneys[Title/Abstract] OR "renal function"[Title/Abstract] OR "renal functioning"[Title/Abstract] OR “renal disease”[Title/Abstract] OR creatinin[Title/Abstract] OR creatinine[Title/Abstract] OR kreatinine[Title/Abstract] OR kreatinin[Title/Abstract] OR GFR[Title/Abstract] OR eGFR[Title/Abstract] OR “glomerular filtration rate”[Title/Abstract] OR MDRD[Title/Abstract] OR Cockroft-Gault[Title/Abstract] OR DMSA[Title/Abstract] OR renography[Title/Abstract] OR renogram[Title/Abstract] OR MAG3[Title/Abstract] OR hydronephrosis[Title/Abstract] OR renal scintigraphy[Title/Abstract] OR “nuclear studies”[Title/Abstract]OR “renal scar”[Title/Abstract] OR “renal scarring”[Title/Abstract] OR “renal damage”[Title/Abstract] OR “upper urinary tract status”[Title/Abstract] OR “upper tract”[Title/Abstract] OR “urinary tract”[Title/Abstract] OR “upper urinary tract”[Title/Abstract] OR “ultrasound”[Title/Abstract] OR hydronephrosis[Title/Abstract]) OR** #5 **(“Vesico-ureteral Reflux”[MeSH] OR VUR[Title/Abstract] OR reflux[Title/Abstract] OR “urinary reflux”[Title/Abstract] OR “urine reflux”[Title/Abstract] OR “renal reflux”[Title/Abstract] OR “vesicoureteral reflux”[Title/Abstract] OR “micturating cystogram”[Title/Abstract] OR cystogram[Title/Abstract] OR cystography[Title/Abstract])**)

**#1 (Spina-synonyms), #2 (incontinence/continence-synonyms), #3 (bladder function), #4 (upper tract function), #5 (reflux-synonyms)**

**#1 AND (#2 OR #3 OR #4 OR #5)**

**RESULTS: 2757**

EMBASE:

#1: **(‘spina bifida’:ti:ab OR ‘spinal dysraphism’:ti:ab OR myelomeningocele:ti:ab OR SB:ti:ab OR meningomyelocele:ti:ab OR MMC:ti:ab OR meningocele:ti:ab OR ‘meningocystocele’:ti:ab OR ‘bifid spine’:ti:ab OR ‘spinal bifida’:ti:ab OR ‘neural tube defects’:ti:ab OR myelodysplasia:ti:ab NOT (‘migrating motor complex’:ti:ab OR mitomycine:ti:ab OR mitomycin:ti:ab OR ‘bone marrow’:ti:ab OR haematology:ti:ab OR leukaemia:ti:ab OR ‘folic acid’:ti:ab OR folate:ti:ab))** **AND (** #2 **(‘urinary incontinence’:ti:ab OR ‘incontinence’:ti:ab OR ‘enuresis’:ti:ab OR ‘urinary loss’:ti:ab OR ‘urine loss’:ti:ab continence:ti:ab OR ‘urinary continence’:ti:ab OR dry:ti:ab OR continent:ti:ab OR incontinent:ti:ab OR dribbling:ti:ab OR dribble:ti:ab OR dryness:ti:ab OR wet:ti:ab OR wetting:ti:ab OR bed-wetting:ti:ab) OR** #3 **(‘lower urinary tract’:ti:ab OR ‘bladder function’:ti:ab OR ‘bladder capacity’:ti:ab OR urodynamics:ti:ab OR ‘urodynamic’:ti:ab OR ‘bladder pressure’:ti:ab OR ‘leak point pressure’:ti:ab OR ‘bladder pressure’:ti:ab OR ‘intravesical pressure’:ti:ab OR ‘intravesical’:ti:ab OR vesical:ti:ab OR ‘detrusor sphincter dyssynergia’:ti:ab OR ‘dyssynergia’:ti:ab OR ‘dyssynergic’:ti:ab OR bladder:ti:ab OR ‘urinary bladder’:ti:ab OR ‘pressure flow studies’:ti:ab OR ‘pressure flow study’:ti:ab OR PVR:ti:ab OR ‘post void residual’:ti:ab OR ‘post void residue’:ti:ab OR ‘post-voiding residue’:ti:ab) OR** #4 **(kidney:ti:ab OR CKD:ti:ab OR ESRD:ti:ab OR dialysis:ti:ab OR ‘renal replacement’:ti:ab OR ‘renal substitution’:ti:ab OR ‘chronic kidney disease’:ti:ab OR renal:ti:ab OR kidneys:ti:ab OR ‘renal function’:ti:ab OR ‘renal functioning’:ti:ab OR ‘renal disease’:ti:ab OR creatinin:ti:ab OR creatinine:ti:ab OR kreatinine:ti:ab OR kreatinin:ti:ab OR GFR:ti:ab OR eGFR:ti:ab OR ‘glomerular filtration rate’:ti:ab OR MDRD:ti:ab OR Cockroft-Gault:ti:ab OR DMSA:ti:ab OR renography:ti:ab OR renogram:ti:ab OR MAG3:ti:ab OR hydronephrosis:ti:ab OR renal scintigraphy:ti:ab OR ‘nuclear studies’:ti:ab OR ‘renal scar’:ti:ab OR ‘renal scarring’:ti:ab OR ‘renal damage’:ti:ab OR ‘upper urinary tract status’:ti:ab OR ‘upper tract’:ti:ab OR ‘urinary tract’:ti:ab OR ‘upper urinary tract’:ti:ab OR ‘ultrasound’:ti:ab OR hydronephrosis:ti:ab) OR** #5 (**VUR:ti:ab OR reflux:ti:ab OR ‘urinary reflux’:ti:ab OR ‘urine reflux’:ti:ab OR ‘renal reflux’:ti:ab OR ‘vesicoureteral reflux’:ti:ab OR ‘micturating cystogram’:ti:ab OR cystogram:ti:ab OR cystography:ti:ab)**

**#1 AND (#2 OR #3 OR #4 OR #5)**
